# Anthracyclines Strike Back: Rediscovering Non-Pegylated Liposomal Doxorubicin in Current Therapeutic Scenarios of Breast Cancer

**DOI:** 10.3390/cancers13174421

**Published:** 2021-09-01

**Authors:** Francesco Schettini, Mario Giuliano, Matteo Lambertini, Rupert Bartsch, David James Pinato, Concetta Elisa Onesti, Nadia Harbeck, Diana Lüftner, Sylvie Rottey, Peter A. van Dam, Khalil Zaman, Giorgio Mustacchi, Joseph Gligorov, Ahmad Awada, Mario Campone, Hans Wildiers, Alessandra Gennari, Vivianne C. G. Tjan-Heijnen, Javier Cortes, Mariavittoria Locci, Ida Paris, Lucia Del Mastro, Sabino De Placido, Miguel Martín, Guy Jerusalem, Sergio Venturini, Giuseppe Curigliano, Daniele Generali

**Affiliations:** 1 Translational Genomics and Targeted Therapies in Solid Tumors Research Group, 08036 Barcelona, Spain; schettini@clinic.cat; 2 Department of Medical Oncology, Hospital Clinic of Barcelona, 08036 Barcelona, Spain; 3 Department of Clinical Medicine and Surgery, University of Naples Federico II, 80131 Naples, Italy; m.giuliano@unina.it (M.G.); deplacid@unina.it (S.D.P.); 4 Department of Internal Medicine and Medical Specialties (DiMI), School of Medicine, University of Genova, 16132 Genova, Italy; matteo.lambertini@unige.it (M.L.); lucia.delmastro@hsanmartino.it (L.D.M.); 5 Department of Medical Oncology, U.O.C Clinica di Oncologia Medica, IRCCS Ospedale Policlinico San Martino, 16132 Genova, Italy; 6 Division of Oncology, Department of Medicine 1, Medical University of Vienna, 1090 Vienna, Austria; rupert.bartsch@meduniwien.ac.at; 7 Division of Cancer, Department of Surgery and Cancer, Imperial College London, London SW7 2AZ, UK; david.pinato@imperial.ac.uk; 8 Department of Translational Medicine, Università del Piemonte Orientale “A. Avogadro”, 28100 Novara, Italy; alessandra.gennari@uniupo.it; 9 Clinical and Oncological Research Department, IRCCS Regina Elena National Cancer Institute, 00144 Rome, Italy; elisaonesti@gmail.com; 10 Breast Center, Department OB&GYN and CCCLMU, LMU University Hospital, 81377 Munich, Germany; nadia.harbeck@med.uni-muenchen.de; 11 Department of Hematology, Oncology and Tumor Immunology, Charité—Universitätsmedizin Berlin, 10117 Berlin, Germany; diana.lueftner@charite.de; 12 Department of Medical Oncology, UZ Gent, 9000 Gent, Belgium; sylvie.rottey@ugent.be; 13 Oncology Department, University Hospital Antwerp (UZA), 2650 Edegem, Belgium; Peter.VanDam@uza.be; 14 Oncology Department, Lausanne University Hospital CHUV, 1011 Lausanne, Switzerland; khalil.zaman@chuv.ch; 15 Division of Medical Oncology, University of Trieste, 34127 Trieste, Italy; mail@mustacchioncology.it; 16 Department of Medical Oncology, Tenon Hospital, Institut Universitaire de Cancérologie AP-HP, Sorbonne University, 75004 Paris, France; joseph.gligorov@tnn.aphp.fr; 17 Department of Medical Oncology, Institut Jules Bordet, Université Libre de Bruxelles, 1000 Bruxelles, Belgium; ahmad.awada@bordet.be; 18 Division of Medical Oncology, Institut de Cancérologie de l’Ouest-Pays de la Loire, 44800 Saint-Herblain, France; mario.campone@ico.unicancer.fr; 19 Department of General Medical Oncology, University Hospital Leuven, 3000 Leuven, Belgium; hans.wildiers@uzleuven.be; 20 Division of Medical Oncology, Maastricht University Medical Center (MUMC), 6229 Maastricht, The Netherlands; vcg.tjan.heijnen@mumc.nl; 21 Oncology Department, IOB Institute of Oncology, Quiron Group, 08023 Madrid, Spain; jacortes@vhio.net; 22 Vall d’Hebron Institute of Oncology (VHIO), Centro Cellex, 08035 Carrer de Natzaret, Spain; 23 Department of Neuroscience, Reproductive Medicine, Odontostomatology, University of Naples Federico II, 80131 Naples, Italy; mariavittorialocci@virgilio.it; 24 Department of Woman and Child Health and Public Health, Woman Health Area, Fondazione Policlinico Universitario A, Gemelli IRCCS, 00168 Rome, Italy; ida.paris@policlinicogemelli.it; 25 Departamento de Medicina, Instituto de Investigación Sanitaria Gregorio Marañón Universidad Complutense, 28007 Madrid, Spain; mmartin@geicam.org; 26 Division of Medical Oncology, CHU Sart Tilman Liège and University of Liège, 4000 Liège, Belgium; g.jerusalem@chuliege.be; 27 Management Department, University of Turin, 10124 Torino, Italy; sergio.venturini@unito.it; 28 Istituto Europeo di Oncologia, IRCCS ed Università di Milano, 20141 Milano, Italy; giuseppe.curigliano@ieo.it; 29 Department of Medicine, Surgery and Health Sciences, University of Trieste, 34127 Trieste, Italy; 30 Multidisciplinary Unit of Breast Pathology and Translational Research, Cremona Hospital, Viale Concordia 1, 26100 Cremona, Italy

**Keywords:** anthracyclines, breast cancer, triple negative, hormone receptor, metastatic, non-pegylated liposomal doxorubicin

## Abstract

**Simple Summary:**

Anthracyclines are among the most active chemotherapies in breast cancer (BC). However, they can cause structural and cumulative dose-related cardiac damage; hence, they require careful administration after preliminary functional cardiac assessment and subsequent monitoring, along with a limitation in the cumulative dose delivered. Non-pegylated liposomal doxorubicin (NPLD) has been precisely developed to optimize the doxorubicin toxicity profile, while retaining its therapeutic efficacy, thanks to a reduced diffusion in normal tissues with preserved drug penetrance into cancer sites. This has allowed administration of NPLD beyond a conventional doxorubicin maximum cumulative dose, as well as in patients with cardiac comorbilities or anthracycline pretreatment. At present, NPLD is approved in Europe and Canada in combination with cyclophosphamide as the first line of metastatic HER2-negative BC. However, given the increasing complexity of the therapeutic scenario in this setting, we have carefully revised the most updated literature on the topic and dissected the potential role of NPLD in the evolving therapeutic algorithms.

**Abstract:**

Anthracyclines are among the most active chemotherapies (CT) in breast cancer (BC). However, cardiotoxicity is a risk and peculiar side effect that has been limiting their use in clinical practice, especially after the introduction of taxanes. Non-pegylated liposomal doxorubicin (NPLD) has been developed to optimize the toxicity profile induced by anthracyclines, while maintaining its unquestionable therapeutic index, thanks to its delivering characteristics that increase its diffusion in tumor tissues and reduce it in normal tissues. This feature allows NPLD to be safely administered beyond the standard doxorubicin maximum cumulative dose of 450–480 mg/m^2^. Following three pivotal first-line phase III trials in HER2-negative metastatic BC (MBC), this drug was finally approved in combination with cyclophosphamide in this specific setting. Given the increasing complexity of the therapeutic scenario of HER2-negative MBC, we have carefully revised the most updated literature on the topic and dissected the potential role of NPLD in the evolving therapeutic algorithms.

## 1. Introduction

Anthracyclines are among the most active chemotherapeutic agents in breast cancer (BC), along with taxanes. Nevertheless, a risk and peculiar side effect induced by anthracyclines is cardiotoxicity, potentially leading to congestive heart failure (CHF) [1]. The specific mechanism underlying this adverse event is still unclear, although it is recognized that a combination of factors represented by the induction of reactive oxygen species (ROS)-mediated oxidative stress; DNA damage; senescence; and cell death pathways activation in cardiomyocytes and, as additional targets, cardiac progenitor cells, cardiac fibroblasts, and endothelial cells, seem to concurrently be responsible [1].

The structural myocardial damage is now thought to have already occurred at the time of first exposure, progressively accumulating over time, and therefore is dose related and substantially irreversible [1]. For this reason, treatment with standard anthracyclines requires continuous periodical cardiac function monitoring, usually with seriated echocardiographies [1]. This examination allows for a non-invasive and accurate evaluation of the left ventricular ejection fraction (LVEF), but also newer parameters that seem to be able to detect early subclinical forms of cardiotoxicity that are still not causing symptomatic or asymptomatic LVEF reductions (e.g., 2D global longitudinal strain, E/e’ ratio, 3D ejection fraction, etc.) [2,3]. It is also necessary to limit the total cumulative dose administered over time [1]. In fact, the cumulative dose recommended by current guidelines is 450–480 mg/m^2^ for standard doxorubicin and 900 mg/m^2^ for the less cardiotoxic epirubicin, although the latter needs to be administered at higher doses as compared with doxorubicin, to obtain similar efficacy (epirubicin 90 mg/m^2^ is considered to be equivalent to doxorubicin 60 mg/m^2^) [4,5,6]. Yet, no absolute safe dose of anthracyclines exists, since cardiotoxicity is a stochastic effect [7].

Given the unquestionable efficacy of anthracyclines in BC and the need to optimize their toxicity profile, the last three decades have seen the development of new mechanisms of drug delivery and their application to this drug class, among others [8]. In addition, concomitant administration with cardioprotective agents such as dexrazoxane have been investigated [9]. Dexrazoxane has been shown to reduce the risk of clinical heart failure and cardiac events in patients with breast cancer undergoing anthracycline chemotherapy without altering the impact on breast cancer outcomes [9]. However, the quality of available evidence is low and dedicated randomized trials are warranted. Moreover, some randomized controlled trials (RCT) in patients with solid tumors treated with anthracyclines found that beta-blockers, such as carvedilol or nebivolol; aldosterone antagonist spironolactone; angiotensin-converting enzyme inhibitors (ACEI); and angiotensin receptor blockers (ARB), such as enalapril or telmisartan, respectively, were associated with a cardioprotective effect. On average, there has been no, or limited, LVEF reduction as compared with the controls in all studies [1]. However, no standardized pharmacologic preventive protocol has been established so far and uncertainties exist on whether to adopt a prophylactic pharmacological approach before, or early after, the onset of subclinical cardiac damage (e.g., subclinical rise in troponins) [1].

An effective way of reducing anthracycline-induced cardiotoxicity that has successfully entered the clinical practice scenario is liposomal encapsulation, with or without pegylation. The pegylated liposomal form of doxorubicin (PLD) is currently approved as a single agent in metastatic BC (MBC) at high risk of cardiotoxicity and shows some differences in the pharmacokinetic and pharmacodynamic profiles as compared with the non-pegylated liposomal formulation [10]. These distinctive features are at the basis of a more delayed administration schedule and some differences in the toxicity profile. Namely, the pegylated form, as compared with the non-pegylated form, has been associated with less frequent high-grade alopecia but higher rates of hand-foot syndrome, which is a dose-limiting toxicity for PLD [11,12]. However, within this paper, we will specifically focus on non-pegylated liposomal doxorubicin (NPLD), with the aim of dissecting its differences with conventional anthracyclines, and highlighting its relevance and optimal positioning in the current therapeutic algorithms.

## 2. Structure and Mechanisms of Action

**Figure 1:**
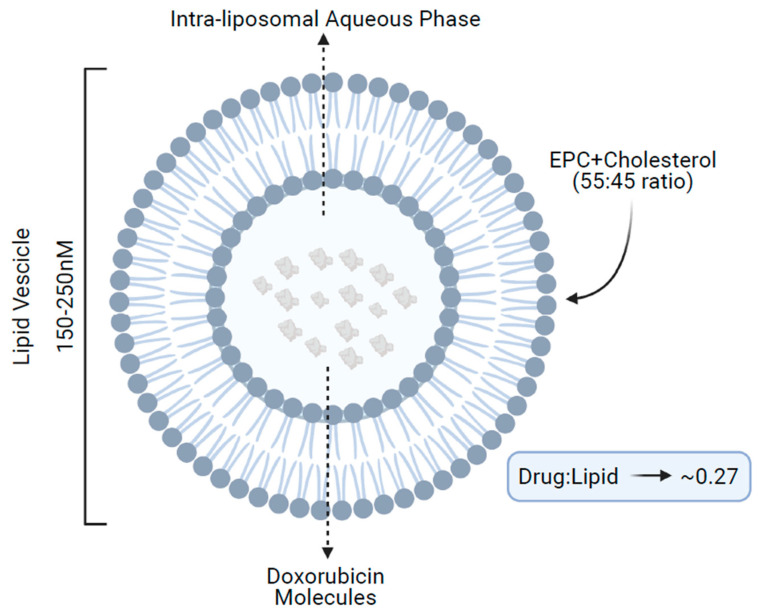
Structure of non-pegylated liposomal doxorubicin. EPC, acidic egg phosphatidylcholine.

NPLD structurally consists of a spherical vesicle of 150–250 nM formed by a lipidic bilayer of acidic egg phosphatidylcholine and cholesterol (55:45 ratio), enclosing an aqueous core, which contains multiple molecules of doxorubicin in a lipid/drug ratio of approximately 0.27 (Figure 1) [13].

Within the core, doxorubicin is protonated, resulting in an entrapment efficacy of the negatively charged lipidic bilayer of more than 99%. This translates into lower levels of free drug in the blood and less nonspecific bindings as compared with conventional doxorubicin. Concurrently, the large size of the liposome vesicles also minimizes doxorubicin exposure to normal tissues, because healthy tissues such as the heart and gastrointestinal tract, do not present the leaky vasculature found at tumor sites. Hence, normal organs are subjected to lower levels of doxorubicin accumulation, leading to a reduced incidence of side effects, including cardiotoxicity. Additionally, the larger size of the liposomes makes them easily recognized by mononuclear phagocytes; in fact, several in vivo preclinical studies have demonstrated that the behavior of the liposomal-encapsulated drug is largely controlled by the interaction of liposomes with the reticuloendothelial system [14,15]. All these features, taken together, are responsible for a differential pharmacokinetic and pharmacodynamic profile between conventional doxorubicin and NPLD, which is on the basis of NPLD performance in clinical trials, as subsequently discussed.

## 3. Current Therapeutic Indication, Efficacy, and Toxicity Profile

NPLD is currently approved in Europe and Canada at a dose of 60–75 mg/m^2^ intravenously (IV) every 3 weeks (q3w), in combination with cyclophosphamide at 600 mg/m^2^, as first-line chemotherapy (CT) for HER2-negative (neg) MBC [13]. This indication is based on the results of three randomized phase III trials [16,17,18]. The first trail was a randomized study of NPLD + cyclophosphamide vs. doxorubicin + cyclophosphamide (AC) as first-line in HER2-negative MBC. The experimental regimen showed comparable response rates (43% vs. 43%), similar time-to-progression (TTP) (median TTP [mTTP] of 5.1 vs. 5.5 months, *p* = 0.82), and overall survival (OS) (median OS [mOS] 19 vs. 16 months, *p* = 0.21), with significantly less cardiotoxicity (6% vs. 21%, reduction in LVEF, including five cases of CHF), neutropenia, need for red blood cells (RBC) transfusions, and mucositis/stomatitis as compared with the control [16]. Interestingly, the NPLD-based regimen was superior to AC in the small subset of patients pretreated with anthracyclines in an early setting in terms of response rates (50% vs. 20%) [16].

Another pivotal trial of NPLD + cyclophosphamide vs. epirubicin + cyclophosphamide (EC) showed consistent results, with NPLD + cyclophosphamide being non-inferior in terms of response rates (46% vs. 39%, *p*_non-inf._ = 0.002), significantly superior in TTP (mTTP of 7.7 vs. 5.6 months, *p* = 0.022), and comparable in terms of OS (mOS of 18.3 vs. 16.0 months, *p* = 0.504) with respect to EC [17]. This is in line with the results of an older study from the French Epirubicin Study Group, which demonstrated that anthracyclines used at higher doses and longer duration did not induce a significant OS benefit, but had a positive impact on response rates and TTP [19]. Neutropenia was more frequent with NPLD but the overall toxicity profile was very similar, unexpectedly including cardiotoxicity rates (12% vs. 10% of LVEF reductions, with no CHF). This might depend on a combination of factors, including the higher dose of NPLD chosen for this trial as compared with the former trial (75 mg/m^2^ vs. 60 mg/m^2^), the lack of statistical power to specifically detect significant differences in cardiotoxicity, and the lower dose of epirubicin as compared with a standard EC regimen.

NPLD was also compared as a single agent to doxorubicin in a phase III RCT where response rates, TTP, OS, and toxicities were found to be similar between the two drugs [18]. However, this trial confirmed the lower cardiotoxicity rates with the liposomal formulation (13% vs. 29% LVEF reductions, including two and nine CHF events, respectively), further supporting its use in clinical practice as a replacement for standard anthracyclines. The main trials’ characteristics and results are detailed in Table 1.

All pivotal trials showed a non-cardiac toxicity profile substantially comparable with that of conventional doxorubicin, with hematologic toxicities (e.g., leukopenia, neutropenia, and anemia), alopecia, diarrhea, nausea/vomiting, and stomatitis/mucositis being the most frequent adverse events. The most frequent (>2% cases within each study) grade 3–5 toxicities were arthralgias, diarrhea, fatigue, febrile neutropenia, nausea/vomiting, stomatitis, leukopenia, neutropenia, anemia, thrombocytopenia, and grade 2 alopecia [16,17,18]. The minimum-maximum rates of grade 3–5 toxicities across pivotal trials are reported in Table 2. Importantly, only one toxic death was reported with NPLD in pivotal trials [16,17,18].

A Cochrane meta-analysis, in 2010, confirmed a significantly lower rate of both CHF and clinical-subclinical heart failure with liposomal-encapsulated doxorubicin (relative risk [RR] 0.20, 95% confidence interval [CI] 0.05–0.75, and RR 0.38, 95% CI 0.24–0.59 respectively) as compared with conventional anthracyclines in the metastatic setting [9]. Another recent meta-analysis, also including the RCT of the alternative pegylated liposomal formulation, confirmed similar efficacy in terms of PFS (*p* = 0.12) and OS (*p* = 0.93), with significantly better response rates (odds ratio [OR] 1.25, *p* = 0.03) and cardiotoxicity profile (OR for cardiotoxicity 0.46, *p* = 0.03) as compared with conventional anthracyclines [20].

Overall, the limited cardiotoxicity is, at present, the most valuable feature justifying the use of NPLD in clinical practice.

## 4. Additional Evidence in Metastatic HER2-Negative Disease

Apart from pivotal trials, a phase III RCT was conducted to compare first-line NPLD + cyclophosphamide with NPLD + vinorelbine, further demonstrating the superiority of the former in TTP (*p* = 0.023), with slightly less non-cardiac toxicities and no symptomatic cardiotoxicity in both treatment arms, with a median NPLD administered dose of 420 mg/m^2^ (range 120–660 mg/m^2^) [21]. Additionally, several prospective trials tested NPLD weekly administration, alone or in combination with taxanes. Those studies confirmed the excellent activity (ORR range of 50–79.6%) and efficacy (mTTP range of 10–12.6 months and mOS range of 23–25 months) of this formulation, with no unexpected toxicities and asymptomatic and reversible LVEF decrease in 10–24% of cases, without CHF events [22,23,24].

Unexpectedly, the Myotax single arm phase II trial of NPLD plus docetaxel as first-line therapy of anthracycline-pretreated HER2-negative MBC showed an unexpectedly unfavorable toxicity profile, with 15% of the patients developing CHF and no apparent explication for this outcome [25]. It should be noted that anthracycline-pretreated patients had already been included in other studies, without negatively affecting the cardiotoxicity profile of NPLD [16,18,22,24,26].

Finally, brain metastases are a common devastating evolution in MBC. While local therapy remains the mainstay of treatment, a recent surge of interest in systemic treatment has been observed. While chemotherapeutic agents may pass through the impaired blood-brain barrier at the metastatic site (blood-tumor-barrier, BTB), Lockmann et al. showed that permeability varied between different metastases and between different areas of a single lesion [27]. The concentration of ^14^C-paclitaxel and ^14^C-doxorubicin within metastases was larger than in normal brain tissue, but less than 15% in extracranial lesions. Liposomal encapsulation may improve drug distribution, as liposomes can pass through the gaps in the leaky vasculature of the BTB and accumulate in brain metastases [28]. However, clinical data with regards to the hypothetical activity of NPLD in breast cancer brain metastases is limited, and therefore this concept requires further evaluation.

## 5. The Early-Stage Scenario

Despite showing comparable efficacy with better toxicity than conventional doxorubicin in metastatic setting, NPLD is currently not approved for early disease. The evidence in this setting for HER2-negative breast tumors is limited. A phase I study by Schmid et al. demonstrated that a combination of NPLD with docetaxel and gemcitabine could provide valuable tumor responses (83% clinical response rates) with a manageable toxicity profile in the neoadjuvant setting and defined the regimen doses for a subsequent phase II study [29]. In this trial, a pathologic complete response (pCR) of 17.5% was observed, preceded by a clinical objective response in 80% of the patients, with a substantially manageable and expected toxicity profile. No cardiac toxicities were observed, and the vast majority of patients completed the treatment [30]. Interestingly, a phase II trial of NPLD + cyclophosphamide followed by weekly paclitaxel in elderly patients (>65 years) showed no median LVEF changes during and after the completion of adjuvant therapy (including a time span of more than 2 years), with no unexpected toxicities and a mild toxicity profile, a median time-to-recurrence (TTR) of 33.9 months, and median OS not reached after a median follow-up of 26.5 months (18.6–48.5 months) [31]. It is important to underline that the majority of the patients enrolled presented with clinically high risk of relapse (59% Ki67 >14%, 50% stage III disease, with 72% node positive tumors, 26% triple negative breast cancer (TNBC), and 68% grade 3 tumors) [31]. Other retrospective experiences showed that replacing conventional anthracyclines with NPLD in (neo)adjuvant regimens including taxanes, produced similar results obtained with conventional anthracycline-containing regimens, with no specific cardiotoxicity concerns and otherwise comparable toxicity profiles [32,33].

The only RCT in early-stage disease were the German Breast Group GeparSixto phase II trial and the French GERICO phase II trial in elderly patients. While not specifically asking the question of NLPD as a component of neoadjuvant CT, in the GeparSixto study, an intense CT backbone of 18 cycles of weekly paclitaxel 80 mg/m^2^ in combination with NPLD 20 mg/m^2^ in patients with triple-negative or HER2-positive (+) early-stage BC was administered [34]. Patients with TNBC also received bevacizumab, while simultaneous trastuzumab and lapatinib was administered in HER2+ BC patients. All patients were randomized to weekly carboplatin or control. Addition of carboplatin to an anthracycline/taxane backbone resulted in a significant improvement in pCR rates from 36.9% to 53.2% (*p* = 0.005) in the subset of patients with TNBC, while no benefit was seen in the HER2+ cohort. In the control group, grade 3/4 neutropenia was observed in 27% of participants, and grade 3/4 diarrhoea in 11%; toxicity was significantly increased by the addition of carboplatin (grade 3/4 neutropenia 65%, grade 3/4 anaemia 15%, grade 3/4 thrombocytopenia 14%, and grade 3/4 diarrhoea 17%). This suggests that weekly NPLD at the dose and schedule chosen is active also in early-stage disease, but given the specific trial design and the lack of a standard control group, no definitive conclusion regarding the potential role of NPLD in the neoadjuvant setting can be drawn. In addition, significant treatment-emergent toxicity was noted, especially in the triple-combination arm [34].

The GERICO trial demonstrated the feasibility of an adjuvant CT regimen with NPLD + cyclophosphamide in fit elderly women (median age 75 [min–max: 70–82] years) with HR+/HER2-negative BC, with no cardiotoxicity and toxic deaths observed and no deleterious impact on functional independence [35]. In addition, the ASTER 70s phase 3 RCT in women aged >70, which includes NPLD + cyclophosphamide as a possible adjuvant CT regimen, is ongoing and results are awaited to draw a more definitive conclusion for this specific population [36].

## 6. The HER2+ Disease: A Brief Overview

In HER2+ breast tumors a significant increase in cardiotoxicity rates was observed with anthracyclines and trastuzumab combinations [1]. Therefore, since the cessation of anti-HER2 therapy is generally not recommended in the advanced setting [37,38], anthracyclines are not an adequate therapeutic partner in this setting. Conversely, when indicated, they can be administered before (neo)adjuvant trastuzumab in early disease without inducing additional effects on the risk of cardiotoxicity [37,39]. For this reason, due to the low cardiotoxic profile, some trials are studying the potential role of NPLD in both early and advanced stage HER2+ BC.

In early-stage disease, the best available evidence is substantially represented by three small single arm phase II trials, which showed pCR rates in breast and axilla with sequential or concomitant NPLD + taxanes and anti-HER2-based regimens ranging between 27 and 56.6% [40,41,42]. Low and acceptable rates of cardiotoxicity were observed (i.e., no CHF and asymptomatic small reductions in LVEF in 0.8–41% cases, without need for treatment discontinuation) [40,41,42]. However, it is important to consider that the true benefit of anthracyclines in early-stage HER2+ disease is being questioned following multiple evidence from prospective trials (e.g., BCIRG-006, TRAIN-2, TRYPHAENA, etc.) that suggest comparable outcomes and less toxicity for anthracycline-free (neo)adjuvant taxane-based regimens and HER2 blockade [43].

In the metastatic setting, NPLD was studied in combination with paclitaxel or docetaxel and trastuzumab as first-line therapy for HER2-positive disease in a series of phase I/II trials, highlighting promising therapeutic efficacy with no concerns for increased cardiotoxicity [26,44,45]. This led to a phase III RCT by Baselga et al. comparing NPLD, paclitaxel, and trastuzumab to paclitaxel and trastuzumab. The study failed to demonstrate a significant progression-free survival (PFS) benefit with the addition of NPLD in the overall population (*p* = 0.174). However, a significant improvement in PFS (20.7 vs. 14.0 months, HR 0.68, 95% CI 0.47–0.99) with a trend for better OS was observed in hormone receptor (HR) negative (neg) patients [46]. Although the frequency of adverse events was higher with the NPLD-containing regimen, no significant difference in cardiac toxicity was observed between the two cohorts [46]. Altogether, these results may have supported, at least in HR-neg./HER2+ tumors, the use of NPLD as part of the backbone CT for trastuzumab. However, shortly thereafter, the CLEOPATRA trial showed impressive improvements in PFS and OS, in a first-line setting, with the combination of trastuzumab, pertuzumab, and paclitaxel over trastuzumab and paclitaxel, establishing the anti-HER2 + taxane triplet as the new first-line standard [47,48]. At the same time, the EMILIA trial proved the antibody-drug conjugate T-DM1 as the best second-line option, following significant PFS and OS improvements over lapatinib + capecitabine [48,49]. In both trials, cardiotoxicity was infrequent and mild [47,49]. Due to the impressive survival benefits obtained with such novel anti-HER2 agents, along with the substantial absence of additional cardiotoxicity when adding pertuzumab to a trastuzumab-based scheme or when using T-DM1 instead of lapatinib, the development of a NPLD-based first-line regimen for HER2-positive MBC was somewhat stopped for some years. Only recently, NPLD was combined with T-DM1 in a phase Ib trial in advanced disease after progression from taxanes and trastuzumab-based therapy. Despite absence of concerns related to cardiotoxicity, the study failed to demonstrate any substantial benefit from the addition of NPLD to T-DM1 [50].

## 7. The Positioning of NPLD in the Current Therapeutic Algorithms

### 7.1. Metastatic Setting

As a premise, it should be noted that all major guidelines recommend as upfront treatment for HR+/HER2-negative MBC without visceral crisis an endocrine therapy (ET)-based regimen, while, where available, novel immunotherapy-based combination (i.e., atezolizumab + nab-paclitaxel or pembrolizumab + paclitaxel or nab-paclitaxel or carboplatin + gemcitabine) should be the preferred first-line option for PD-L1 positive (+) TNBC [37,38,51]. A PARP inhibitor (olaparib or talazoparib) might also be a valuable first-line option for HER2-negative *BRCA*1/2-mutant tumors. However, the most appropriate positioning for this drug class in *BRCA*-mutant HR+ disease and PD-L1+ TNBC still remains uncertain [37,38,51,52]. This is especially true for HR+ BC, where novel ET + CDK4/6-inhibitors regimens have demonstrated impressive OS improvements, which are likely to also be retained in BRCA-mut cases [53,54]. In the case of *BRCA*1/2-wild type/PD-L1 negative TNBC, no tailored target therapies are available, and no optimal first-line CT has been established so far. Similarly, in endocrine refractory HR+/HER2-negative tumors, CT becomes the treatment of choice, with no specific recommended regimen [37,38,51].

Due to the substantially palliative role of CT in the metastatic setting, the lack of an established optimal first-line CT, and no unquestionable superiority of poly-CT regimens over mono-CT, all major treatment guidelines recommend a monotherapy to mitigate treatment toxicities, unless a more rapid tumor response was required for clinical reasons. In this case a combination regimen should be the preferred option [37,38,51]. Because no single agent has demonstrated a clear superiority over the others and the evidence for efficacy are strongest for taxanes and anthracyclines, guidelines usually suggest the use of upfront anthracycline and/or taxane-based regimens. In any case, treatment selection should be based on previous therapy, time to recurrence, differential toxicity, comorbid conditions, and patient preferences [37,38,51].

According to the evidence that we have previously discussed, taking together all recommendations from major guidelines and regulatory approval specifications, we hereby provide several considerations regarding the optimal NPLD positioning in the CT-based metastatic therapeutic algorithm for HER2-negative MBC.

In anthracycline-pretreated patients a taxane is usually the preferred option. However, there are several reasons which make NPLD + cyclophosphamide a valuable first-line option in this subset of patients, when a doublet is required:

1. The efficacy of NPLD in anthracycline-pretreated patients has been demonstrated in several trials [16,17,18,55].
2. The reduced cardiotoxicity potentially allows clinicians to administer higher cumulative doses of doxorubicin if the liposomal formulation is adopted.
3. The prescription caveats that limit the use of NPLD + cyclophosphamide to first-line settings and the possibility to still provide patients with very effective taxane-based regimens in the second and subsequent lines (i.e., nab-paclitaxel, paclitaxel, and docetaxel), if not used in the first-line setting.

A similar reasoning can be applied to taxane-pretreated patients and anthracycline/taxane-naïve patients, for whom the potentially better first-line therapeutic options might be either an anthracycline-based or a taxane-based regimen (nab-paclitaxel in taxane-pretreated). However, taxanes are effective and prescriptible also in further lines and NPLD + cyclophosphamide is equally effective but less cardiotoxic than conventional anthracycline-based regimens, with the potential to be administered for even longer periods in responding naïve patients.

In patients that have already received anthracycline/taxane-based regimens, other options include liposomal doxorubicin formulations, capecitabine, gemcitabine, platinum-based compounds, eribulin, vinorelbine, nab-paclitaxel (effective also in taxane-pretreated patients), and ixabepilone (not approved in Europe). In addition, we would like to support our previous reflections with unpublished results from a previous Bayesian network meta-analysis from our group, where all available ET and CT ± target therapies (TT) for first/second-line HR+/HER2-negative MBC were compared [11]. A treatment ranking based on the PFS/TTP results was obtained based on the evaluation of the surface under the cumulative ranking (SUCRA) values [56]. When excluding from the ranking all CT regimens that were not approved for clinical practice and the ET-based regimens, NPLD + cyclophosphamide was among the top 10 CT options in terms of efficacy. More specifically, it was ranked eighth, after doxorubicin + docetaxel (AD), paclitaxel + bevacizumab, capecitabine, doxorubicin + paclitaxel (AT), docetaxel + capecitabine, AC, and eribulin. It should be considered that the meta-analysis could not be performed according to separate first-line or second-line settings. Therefore, it is highly likely that the capecitabine and eribulin results could be ranked higher than NPLD + cyclophosphamide because their efficacy in second/further line trials, their most common setting of use, boosted their performance. At the same time, anthracycline + taxane concomitant combination schemes, despite being highly effective, are quite toxic and burdened by the usual cardiotoxicity issues related to conventional anthracyclines. Considering that CT for MBC is palliative and should be preferably administered until tumor progression or unacceptable toxicity, the higher cumulative dose of NPLD that is potentially deliverable could drive the balance towards its use instead of conventional anthracycline formulations. In this perspective, the first-line combination of NPLD and nab-paclitaxel for HER2-negative MBC has been proven to be sufficiently safe with encouraging efficacy results [23]. The advantage of the combination with nab-paclitaxel over other taxanes, resides in the weekly schedule that can ameliorate the tolerability of the two combined drugs. Moreover, nab-paclitaxel has been demonstrated to be more effective than standard three-weekly paclitaxel and docetaxel, and also effective in taxane-pretreated patients, with an overall acceptable and manageable toxicity profile, despite high rates of peripheric neuropathy, reduced in the case of 100–125 mg/m^2^ weekly schedules [57,58,59]. Therefore, a further development of this combination regimen in patients that already received anthracyclines and taxanes in the early setting might be envisioned, especially in PD-L1 negative TNBC and BRCA-wild type HER2-negative BC, even more in cases of high tumor burden and when a rapid tumor shrinkage is required.

**Figure 2:**
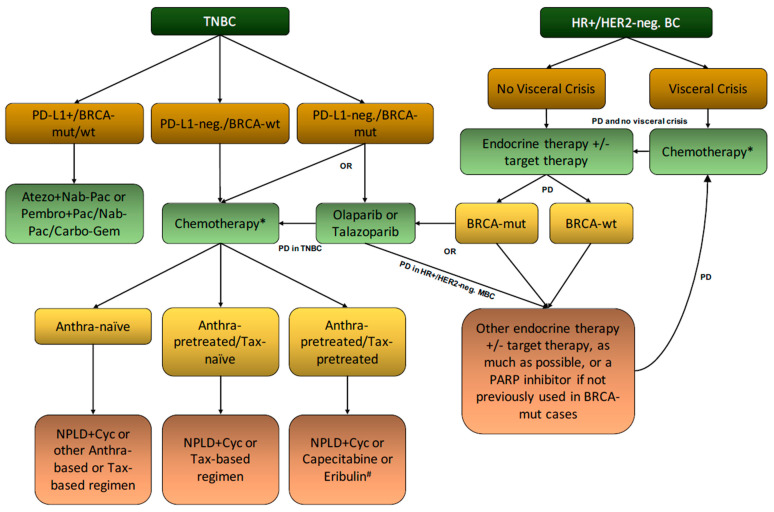
Proposed positioning of NPLD in the current therapeutic algorithm of HER2-negative MBC. NBC, triple negative breast cancer; BC, breast cancer; HR, hormone receptor; +: positive; PD, progression of the disease; mut, mutant; wt, wild-type; NPLD, non-pegylated liposomal doxorubicin; Cyc, cyclophosphamide; Anthra, anthracyclines; Tax, taxanes; neg., negative; ET, endocrine therapy; Atezo, atezolizumab; Pembro, pembrolizumab; Nab-Pac, nab-paclitaxel; Pac, paclitaxel; Carbo-Gem, carboplatin + gemcitabine; *, same as here; #, capecitabine and eribulin are approved as first-line option only for Anthra- and Tax-pretreated patients or in case of specific Anthra and Tax contraindication.

To conclude, based on the previous evidence-based reasoning and prescription caveats, we unanimously achieved a consensus regarding the potentially optimal NPLD positioning in the therapeutic algorithm of HER2-negative MBC, which is schematically reported in Figure 2.

### 7.2. Early-Stage Setting, Elderly Patients, and Main Limitations

Concerning the potential use in the (neo)adjuvant setting in both HER2-negative and positive disease, it must be taken into consideration that the available evidence suggests a potentially similar efficacy to doxorubicin, with acceptable response rates, but high-quality evidence is limited and long-term outcomes have not been properly evaluated in this setting. This is particularly relevant, since the importance of apparent differences in tissue distribution between NPLD and conventional doxorubicin might lead to a reduced efficacy in the control of micrometastases. Therefore, we would recommend caution in substituting tout court conventional anthracyclines with NPLD in early-stage BC. At the same time, NPLD might be a valuable therapeutic option to be considered for those patients at moderate/high risk of relapse, which in principle should receive an anthracycline + taxane-based (neo)adjuvant CT, but who are not eligible for conventional anthracyclines due to the presence of cardiac comorbidities. In this perspective, reassuring data from several lymphoma studies highlighted the safety of NPLD in the case of pre-existing cardiac comorbilities [60,61,62,63]. Similarly, in elderly patients, where hypertension, diabetes, coronary artery disease, and cardiac dysfunction (which are all risk factor for anthracyclines’ cardiotoxicity) are more frequently present than in younger patients, NPLD might be an option to consider, as also recommended by the International Society for Geriatric Oncology [64]. In all these cases, the use of NPLD (which would be off-label in early-stage disease) might be considered after a careful preliminary cardiologic assessment and the absence of symptomatic reduced LVEF.

Importantly, the higher costs of NPLD over doxorubicin (a factor of 100 approximately) and epirubicin (a factor of 4) might represent an additional limitation to a broader NPLD use, independently from the disease scenario. However, it is worth considering that, apart from acquisition costs, no published pharmacoeconomic data are available in terms of direct and indirect costs, such as cardiac events/cardiac heart failure prevented, life-year saved, and treatment-associated costs, to adequately compare the two types of drugs.

Finally, it is also important to consider that NPLD is more difficult than standard doxorubicin to prepare for injection, thus, it is more time-consuming and requires more trained personnel.

### 7.3. Cardiac Monitoring

It is important to take into consideration that the evaluation of LVEF with multiple-gated arteriography (MUGA) or echocardiography (ECHO) is considered to be mandatory before the start of treatment and at each additional administration of NPLD once a patient exceeds a lifetime cumulative anthracycline dose of 550 mg/m^2^ or whenever cardiomyopathy is suspected [63]. All patients receiving NPLD, in general, should routinely (e.g., every 3 months) undergo ECG and MUGA/ECHO monitoring [1,65]. Furthermore, periodical detection of troponins to identify patients with subclinical cardiotoxicity and, in case, starting early treatment with ACE-inhibitors or β-blocker agents to prevent anthracycline-related left ventricular dysfunction and cardiac events are also potentially useful strategies, though not uniformly adopted in current clinical practice [1,66].

## 8. Conclusions

NPLD + cyclophosphamide is currently approved as a first-line CT for HER2-negative MBC. NPLD is significantly less cardiotoxic than common anthracyclines and is also effective in anthracycline-pretreated patients, but the approved combination is more toxic than most common mono-CT available for the treatment of MBC. Therefore, based on the current evidence, NPLD might be a good therapeutic solution when poly-CT is the preferred option (e.g., need for rapid tumor shrinkage). In this case, the best candidates might be patients that already received anthracyclines and/or taxanes for early-stage disease and/or for whom an anthracycline-based regimen might be indicated (e.g., taxane-pretreated or not tolerated) but present with controlled cardiac comorbilities. Moreover, patients should be affected by either PD-L1 negative/*BRCA*-wild type TNBC or *BRCA*-wild type HR+/HER2-negative BC, where immunotherapy-based combinations and PARP inhibitors are not viable options (Figure 2).

The combination with nab-paclitaxel in a weekly schedule is also promising in the first-line setting, and therefore merits further investigation. Conversely, caution should be exercised for substituting conventional anthracyclines with NPLD in early disease, because of a possible limited efficacy in eradicating micrometastases, due to a reduced distribution in normal tissues. Nevertheless, elderly patients and patients with controlled cardiac comorbilities candidates for anthracycline-containing (neo)adjuvant CT, might be an ideal target population. Finally, NPLD can be administered beyond reaching a lifetime cumulative anthracycline dose of 550 mg/m^2^, but the evaluation of LVEF is considered to be mandatory before each additional administration, as well as whenever cardiomyopathy is suspected.

## Figures and Tables

**Table 1:** Main characteristics of phase III pivotal trials of NPLD.

Study Characteristics	Trial 1	Trial 2	Trial 3
*First Author*	Batist G	Harris L	Chan S
*Year*	2001	2002	2004
*Journal*	J Clin Oncol	Cancer	Ann Oncol
*Phase*	III	III	III
*Randomization*	Yes	Yes	Yes
*Study population*	HER2-neg. MBC	HER2-neg. MBC	HER2-neg. MBC
*Arm A*	NPLD + Cyc	NPLD	NPLD + Cyc
*Doses Arm A*	60 mg/m^2^ + 600 mg/m^2^	75 mg/m^2^	75 mg/m^2^ + 600 mg/m^2^
*Arm B*	AC	Doxorubicin	EC
*Doses Arm B*	60 mg/m^2^ + 600 mg/m^2^	75 mg/m^2^	75 mg/m^2^ + 600 mg/m^2^
*N Arm A*	142	108	80
*N Arm B*	155	116	80
*RR Arm A*	43% (95% CI: 35–52%)	26% (95% CI: NR)	46% (95% CI: 35–58%)
*RR Arm B*	43% (95% CI: 35–51%)	26% (95% CI: NR)	39% (95% CI: 28–50%)
*mTTP Arm A*	5.10	3.80	7.70
*mTTP Arm B*	5.50	4.30	5.60
*HR (B vs. A)*	1.03	0.92 *	1.52
*P*	0.82	0.59	0.02
*mOS Arm A*	19.00	16.00	18.30
*mOS Arm B*	16.00	20.00	16.00
*HR (B vs. A)*	1.04	0.76 *	1.15
*P*	0.79	0.09	0.50

PLD, non-pegylated liposomal doxorubicin; AC, doxorubicin + cyclophosphamide; EC, epirubicin + cyclophosphamide; Cyc, cyclophosphamide; RR, response rates; CI, confidence interval; mTTP, median time-to-progression; mOS, median overall survival; HR, hazard ratio; neg, negative; NR, not reported; MBC, metastatic breast cancer; * A vs. B.

**Table 2:** Grade ≥3 adverse reactions rates observed in phase III pivotal trials of NPLD + cyclophosphamide.

ADVERSE REACTIONS WHO GRADE ≥3	NPLD + CYC	NPLD
**non-hematologic non-cardiac events**	**% pt**	**% pt**
Allergic reactions	-	4.0
Alopecia *	62.0	-
Arthralgia	-	4.0
Constipation	1.0	-
Diarrhea	1.0–3.0	1.0
Fatigue/asthenia	0.0–6.0	14.0
Febrile neutropenia	5.0–9.0	0.0–11.0
Hand-foot syndrome	-	10.0
Infection	7.0–11.0	5.0
Nausea/vomiting	2.0–21.0	13.0
Skin reaction/rash	0.0	1.0
Stomatitis/mucositis	4.0–7.0	9.0
**Hematologic and biochemical events**		
Decreased hemoglobin/anemia	3.0–25.0	22.0
Thrombocytopenia	2.0–22.0	13.0
Neutropenia	61.0–87.0	50.0
Leukopenia	16.0	-

WHO, World Health Organization; NPLD, non-pegylated liposomal doxorubicin; CYC, cyclophosphamide; pt, patients; *, grade 2 for alopecia as maximum toxicity grade A.

## Data Availability

The original data that were used to generate the treatment ranking mentioned within Section 7.1 are available from the corresponding author upon reasonable request.
